# Assessment of the risk of malaria re-introduction in the Maremma plain (Central Italy) using a multi-factorial approach

**DOI:** 10.1186/1475-2875-11-98

**Published:** 2012-03-30

**Authors:** Roberto Romi, Daniela Boccolini, Roberto Vallorani, Francesco Severini, Luciano Toma, Maurizio Cocchi, Angelo Tamburro, Gianni Messeri, Antonio Crisci, Luca Angeli, Roberto Costantini, Irene Raffaelli, Giorgio Pontuale, Isabelle Thiéry, Annie Landier, Gilbert Le Goff, Anna Maria Fausto, Marco Di Luca

**Affiliations:** 1 Department of Infectious, Parasitic and Immuno-mediated Diseases, Unit of Vector Borne Diseases, Istituto Superiore di Sanità, Viale Regina Elena 299, Rome 00161, Italy; 2 Consorzio LAMMA, Laboratory of Monitoring and Environmental Modelling for Sustainable Development Sesto Fiorentino, Florence, Italy; 3 Operative Unit of Environmental Zoology, AUSL 9, Grosseto, Italy; 4 Institute of Biometeorology, National Research Council, Florence, Italy; 5 Institut Pasteur, Plateforme CEPIA Département de Parasitologie et Mycologie, Paris, France; 6 UMR MIVEGEC, Institut de Recherche pour le Développement, Montpellier, France; 7 IBAF Department, Università della Tuscia, Viterbo, Italy

**Keywords:** Mosquito-borne diseases, Residual anophelism, *Anopheles labranchiae*, Vectorial capacity, Climate change, *Plasmodium falciparum*, Experimental infection

## Background

In recent years, the increase in globalization [1], the rise in the average temperature of the earth together with an increasing frequency and intensity of extreme weather events, as storms, floods and droughts [2,3], and the environmental changes induced by human activities [4], have raised the concern about the possible introduction or reintroduction of Vector Borne Diseases in Countries where these were absent or eradicated [5]. These considerations, coupled with the recent spread of some mosquito vector borne diseases in Europe [6,7] and the increasing number of imported malaria cases recorded in the Continent [8] have renewed interest in the possible reintroduction of malaria in Southern Europe [7-9], particularly in the countries facing the Western Mediterranean Basin, where potential *Anopheline *vectors are still present [10-13]. Moreover, in recent years autochthonous malaria cases have been sporadically reported in Italy, France, Spain and Greece [14-20].

In 2005, a five-year study was implemented in Italy, as well as in other South European countries, with the aim to assess the status of the local potential malaria vectors and the possible re-introduction of malaria transmission [21-25]. In Italy, the selected study area was the Maremma plain, a region that was hyperendemic for malaria until 60 years ago [26-28] and that more recently was recognized as the major "at risk" area for the malaria reintroduction into Italy [14,29,30].

In Maremma, after the malaria eradication campaign (1947-1951), *Anopheles labranchiae*, the main endophilic vector of the *Anopheles maculipennis *complex was dramatically reduced in abundance. However, in subsequent years, the species has progressively re-colonized most of the area coming back to substantial densities [31-33]. This was mainly due to the introduction of intensive rice cultivation in the early 1970s. Since then, Maremma has been subjected to continuous entomological surveillance that was intensified after 1997, when an autochthonous *Plasmodium vivax *malaria case, transmitted by *An. labranchiae*, occurred in the Province of Grosseto [14]. The studies carried out in the area since eradication, provides a database that allowed a follow-up the history of malaria and its vectors in Maremma over the past 60 years. Starting from the findings of the most recent entomological and environmental studies [23,34], the present study was chosen to evaluate the malariogenic potential of the area using a multifactorial approach.

## Methods

### Study design

The risk of malaria reintroduction was evaluated as the "malariogenic potential" of the study area by assessment of the three parameters that define it:

a. receptivity of the area, given by the presence, distribution, seasonal abundance and bionomics of the potential vector;

b. susceptibility of the vector, that is its ability to become infected with *Plasmodium vivax *and *Plasmodium falciparum*;

c. vulnerability of the territory, that is the possible introduction of malaria reservoirs, given by the number of gametocyte carriers able to infect the vector and present in the study area during months favourable to malaria transmission.

Approaches to evaluate these parameters were:

1) Field collection of further entomological data (bionomics, distribution, abundance) for mosquitoes of the *An. maculipennis *complex; 2) investigation of seasonal dynamics of the vector through the implementation of a weather-based statistical dynamic model; 3) production of a distribution/predictive map of *An. labranchiae *across the study area; 4) evaluation of the length of the possible transmission season for *P. vivax *and *P. falciparum*; 5) assessment of the vector competence of the species to *P. falciparum *by artificial infection; 6) evaluation of the vectorial capacity of *An. labranchiae *in the site where the species is most abundant; 7) risk assessment related to the possibility that the vector may feed on gametocyte carriers occasionally circulating in the study area.

### Study area and collection sites

**Figure 1:**
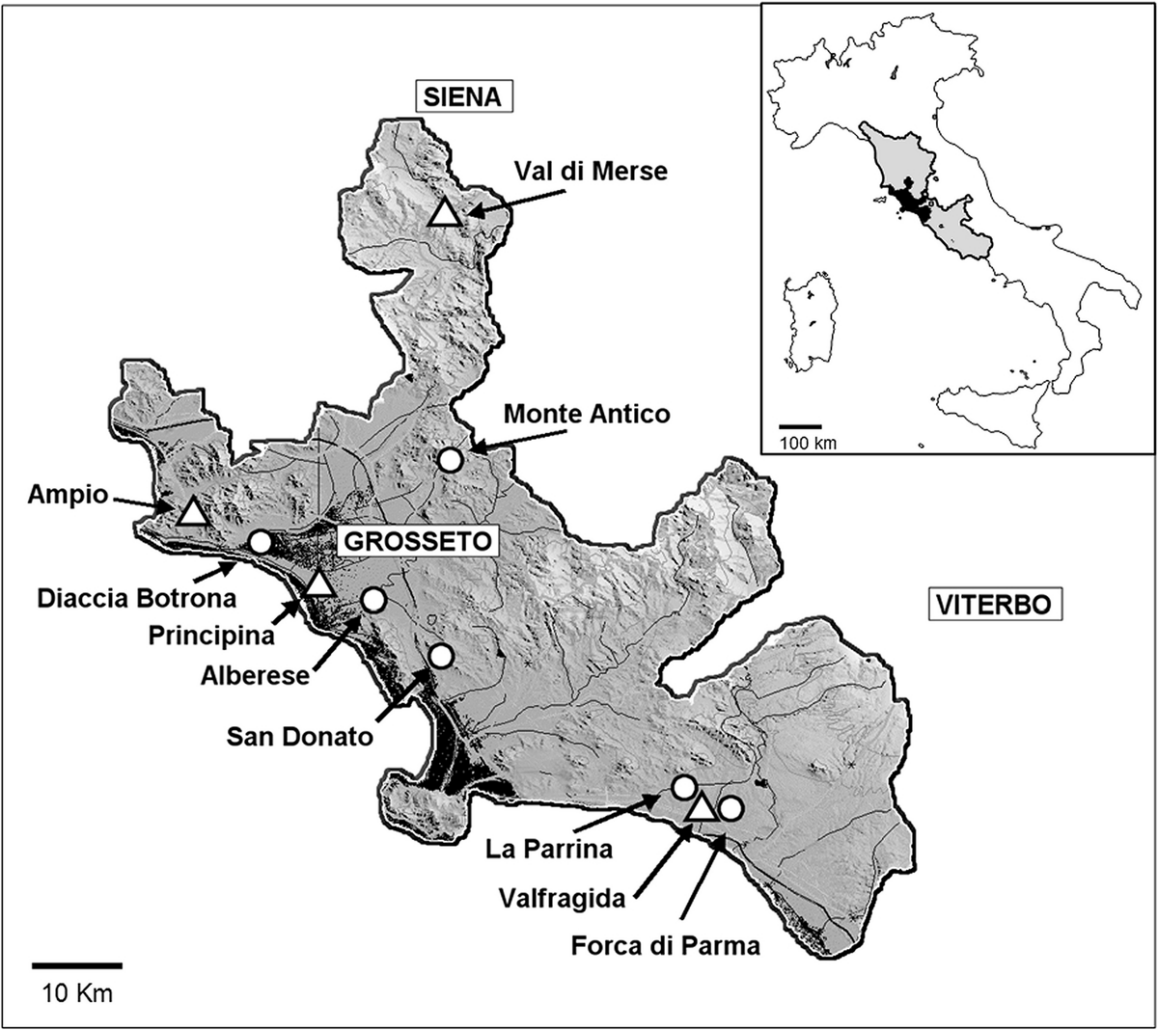
**Map of the study area, the Maremma plain, with localization of the 10 sites considered in the study between 2005 and 2009**. Coastal marshes and the main rivers/streams are in dark grey. Triangles indicate the 4 sites selected for longitudinal surveillance (sites 1,3,4,6 in Table 1) visited fortnightly 6 months/year (May-October). Dots indicate the remaining 6 sites visited sporadically (1-4 times/season, in July-August).

Maremma is a coastal plain of Central Italy, that covers about 5,500 km^2^, extending along the Tyrrhenian coast for about 180 km from mid Tuscany (northern limit 43,3°N) to upper Latium (southern limit 42,17°N). Inland, Maremma extends from the sea to the pre-Apennine foothills, with a width ranging from 16 to 50 km. The territory includes different biotopes where natural and anthropic environments coexist. A wide sandy seashore and dunes, covered by typical Mediterranean bush, gives way to extensive pine woods. Large pastures alternate with intensively cultivated areas up to a range of hills that represents the inner Eastern limit of the study area. About 44,000 ha, nearly 10% of the whole surface of the Maremma plain, is included in protected reservation parks. The study area corresponds to the "core" of this region, shared between the administrative Provinces of Grosseto, and Siena (Tuscany) and Viterbo (Latium) (Figure 1). Ten collection sites were selected on the basis of their environmental characteristics, including three large rice cultivation areas, five rural areas with intensive farming and two protected coastal areas (Table 1).

**Table 1:** Location and features of the sites selected for assessing malaria risk in Maremma in 2005-2009

Site (Province)	Geo-reference	Site features	*An. maculipennis *s.l. range of abundance		*An. labranchiae *prevalence (%)
				
			Larvae (No/dip)	Females (No/shed)	
**1) Principina (GR**)	42°43'29″N 11°02'28″E	Farm: coastal rice-fields, 256 ha*	1-5	> 60 (100-500)	> 96
**2) San Donato (GR**)	42°42'10″N 10°47'15″E	Farm: coastal rice fields, 114 ha*	1-5	> 60 (100-500)	> 98
**3) Val di Merse (SI)**	43°09'11″N 11°17'18″E	Farm: hilly rice fields, 110 ha*	1-5	> 60 (100-500) **(*An. lab*. < 60)**	2
**4) The Ampio (GR)**	42°47'30″N 10°56'51″E	Farm: inner-plain area	0.1-1	< 60 (10-20)	> 98
**5) Monte Antico (GR)**	42°59'11″N 11°21'26″E	Farm: inner-hilly area	0.2-2	< 60 (5-10)	16
**6) Valfragida (VT**)	42°18'26″N 11°39'29″E	Farm: coastal plain area	0.1-1	< 60 (10-40)	85-90
**7) Forca di Parma (VT**)	42°17'88″N 11°42'89″E	Farm: inner plain area	0.5-3	> 60 (20-80)	> 98
**8) La Parrina (VT**)	42°19'20″N 11°38'48″E	Farm: coastal plain area	0.5-3	> 60 (20-80)	> 98
**9) Alberese Natural Park (GR)**	42°49'11″N 10°54'20″E	Coastal Natural area 10,000 ha	0.01-0.05	< 60 (20-30)	> 96
**10) Diaccia Botrona (GR)**	42°54'27″N 10°50'56″E	Brackish water coastal marshes, 236 ha	0.1-2	< 60 (5-10)	> 98
**11) Artificial water collections (GR;SI, VT)**	--	Basins for agricultural, commercial and leisure purposes, 0.1-4.6 ha	0.01-0.1	< 60	--

*Max of rice field surface extension

Sites are distinguished by typology, productivity of *Anopheles maculipennis *s.l. larvae and adults and *An. labranchiae *rate of prevalence within the local population of the complex. Data on both larval density and resting female abundance in animal shelters are reported as a range of seasonal collections

### Mosquito collection and laboratory processing

Collections of *An. maculipennis *s.l. mosquitoes were performed between 2005 and 2009 during 40 surveys. In particular, sites 1, 3, 4 and 6 were visited by 28 fortnightly surveys carried out from April 2005 to October 2006 [23], while in 2007-2009 these sites and the remaining ones were subject to sporadic surveys (1-4 times) in June-August. Mosquito larvae were collected by an enamel standard 500 ml dipper. The number of dipping stations and dips by station was adequate to the type and size of the breeding sites visited according to a standardized protocol [23,29]. Adult collections, using manual or battery-powered aspirators, were mainly targeted at resting females in animal shelters; other kinds of premises, such as haylofts, woodshed, fodder and tools depots represented less than 5% of the premises inspected. A minor fraction of *An. maculipennis *s.l. females were collected by CDC light/CO2 traps. Three night catches on human bait were seasonally carried out in late June, mid July and late August, between 2005 and 2007, respectively in Principina, S. Donato, and Val di Merse. Catches were performed as described in Romi et al. [29]. Mosquitoes were analyzed for species identification, blood meal source and age population structure as described in Di Luca et al. [23]. Previous entomological data used for comparisons are from the Operative Unit of Environmental Zoology, AUSL 9, Grosseto.

### Computing seasonal dynamic of *An. labranchiae *populations

A binomial model was developed for the less productive sites (4 and 6) categorizing the entomological data with the threshold of one adult ("A" absence category/"B" presence category), while the multinomial model, developed for the most productive site 1, was categorize with the thresholds of one and 60 adults ("A" absence category/"B" medium-low presence category/"C" medium-high presence category). The multi-logistic model input were derived from a principal component analysis that leads to an optimal dimensionality reduction of matrix predictors. Akaike Information Criterium (AIC) [35] was finally used for the statistical model parameters selection in order to optimize model performances and to give a better discrimination in the microclimatic variability among different sites. Concerning model selection the statistical function (i.e. STEPAIC) used is taken from MASS R package [36,37]. Weekly model outputs consist essentially on the occurrence probability associated to each adult abundance category, hence the forecasted category was chosen as the one with highest probability. The length of development season was estimated as the number of weeks between the first and the last week with a presence category (B or C category) along the year [38-41]. A verification procedure to assess the reliability of the models was implemented for each site using skill score indexes derived from contingency Tables with observed and forecasted values: the BIAS index, the POD index (Probability Of Detection) and the FAR index (False Alarm Ratio) [42].

### Mapping larval index and adult distribution through geospatial statistical analysis

The quantitative mapping of larvae/adult mosquito presence and abundance was implemented following the approach suggested by Tran *et al. *[43]. The logistic regression model for *Anopheles hyrcanus *in the Camargue region, that explained the presence of larvae as a function of biotope and distance to the nearest rice field, was modified in order to obtain a larval index (probability of observing one larva in a point of a biotope at least once during the mosquito season) consistent with the collection data reported in Table 1. The logistic regression model for *An. labranchiae*, used in this study and implemented with Builder tool from ESRI ArcGIS™, consider also the distance from farm with livestock to the main biotopes as a new explanatory variable in addition to those used by Tran *et al. *Assuming that the abundance of adult mosquitoes is influenced by the presence of breeding sites in the surroundings, the adult index map was derived from the larval index map. Corine Land Cover 2000 (CLC 2000, produced by the European Environment Agency, EEA) spatial data sets for Italy (scale 1:100,000) was used to describe the environmental characteristics likely to influence the spatial distribution of *An. labranchiae*. Also a natural colour aerial photos (May 2007) with a spatial resolution of 1 metre was used to detect the main biotopes where *An. labranchiae *larvae and adults were collected, such as rice fields, reed beds, marshes, temporarily flooded rush wetland and clear water. A photo-interpretation was carried out through a workstation with ESRI ArcGIS™ software. Geographical database in shape-file format was used for localizing livestock and intensive cattle breeding farms in Grosseto Province [44]. The highest abundance of *An. labranchiae *was assumed to be related to rice paddies, and thus the distance to the nearest rice field was computed for each pixel using Geographic Information System (GIS) functionality.

### Evaluation of the length of the possible transmission season

Since Grosseto Airport weather station (42.75°N-11.07°E, 7 m. a.s.l.) is the climate reference centre for the World Meteorological Organization [WMO], meteorological data from that station were considered representative of the whole study area. Thermo-pluviometric diagram with monthly mean temperature and rainfall amount respectively over a 30-years-period (1961-1990) and over the study period 2005-2009 were compared with the minimum temperature for development of *P. vivax *and *P. falciparum*, 15°C and 18°C respectively [45]. The average and absolute values on a yearly and seasonal (May-August) basis of temperature and rainfall were compared for 1961-1990 and 2005-2009 period in order to highlight the possible variation from a climatic point of view (Table 2). An evaluation of the *Plasmodium spp *possible transmission season along the years 2005 and 2006, for the survey period between 2005 and 2009 and for climatological period 1961-1990 was also performed through the Gradient Model Risk (GMR) index calculation [25,37]. GMR consists on the monthly evolution of accumulated values of the index itself to gain insight into possible transmission periods along the year; this index considers only climatic parameters as the minimum mean temperature required for the development of the parasite inside the vector, as reported above, monthly precipitation (mm) and PET (mm.), calculated by the equation:

GDD×R/PET,ifR/PET>0.2

**Table 2:** Most significant climatic parameters for the 1960-1990 and 2005-2009 periods (Grosseto Airport Weather Station)

Climatic Parameters	1961-1990**	2005-2009**	Variation
**Yearly Mean Temperature (°C)**	14.8 (13.9-15.5)	15.6 (14.9-15.8)	0.8
**Yearly Tmin**	9.3 (8.5-10.3)	9.8 (9.1-10.4)	0.5
**Yearly Tmax**	20.2 (19.3-21.2)	21.4 (20.6-21.8)	1.2
**Absolute Tmin/Tmax**	(-13.7/39.2)	(-6.4/+37.8)	--

Seasonal Mean Temperature (°C)*	20.8 (19.3-21.8)	22.0 (21.9-22.5)	1.2
Seasonal Tmin*	14.6 (13.2-15.9)	15.5 (15.1-15.9)	0.9
Seasonal Tmax*	27.0 (25.3-28.1)	28.6 (28.0-29.2)	1.6
**Absolute Tmin/Tmax**	(-0.3/39.2)	(5.8/37.8)	--

**Yearly Mean Rainfall (mm)**	677.7 (422.7-1039.6)	627.4 (402.3-849.3)	-50.3
Seasonal Mean Rainfall (mm)*	126.7 (25.8-316.6)	115.1 (57.2-196.1)	-11.6

*Seasonal data are referred to the 4 months of *Anopheles labranchiae *maximum abundance, (May-August)

**In brackets the extreme values for each period

where GDD is growing degree-days with a base temperature of 15°C and 18°C respectively for *P. vivax *and *P. falciparum*, R is the rainfall and PET is potential evapotranspiration calculated with the empirical method of Thornthwaite 1948 [46] as a function of mean temperature and latitude. The GMR index shows that a transmission risk exists when its value is equal or higher than 116, that is the value required for one *Plasmodium spp *generation.

### Artificial infection assays

Field samples of *An. labranchiae *females, collected in site 1 (Figure 1, Table 1) were submitted to the Plateform CEPIA (Institute Pasteur, Paris, France) to artificial blood infection with gametocyte-containing cultures of the *P. falciparum *NF54 African strain in 2008 and 2009. Production of mature gametocytes and artificial blood infection were performed following procedures described in Mitri *et al. *[47]. A laboratory colony of *Anopheles gambiae *(Ngousso, Cameroon) was used as a positive control. Mosquitoes were dissected on 8^t^and 15 days post-infection to determine prevalence and oocyst load in the midgut. For each experiment all *An. labranchiae *females and the control *An. gambiae *strain were starved 24 hours prior to blood feeding. The infected red blood cells containing *P falciparum *gametocytes complemented with fresh RBC and human AB serum, were deposited in a Parafilm^® ^membrane feeder previously warmed at 37°C. After 15 minutes feeding, unfed *An. labranchiae *were offered a second blood meal on the next day, when possible. Engorged females were kept at 26 ± 1°C inside small cages and were provided with 10% sucrose until dissection 8 days or 15 days post infection. In the 2009, the detection of sporozoites was carried out at the Institute pour la Recherche et le Développement in Montpellier (IRD, France), using the cut head-thorax from the 15^th ^day survived mosquitoes. DNA extraction was performed by a single-round, multiplex PCR, according to Padley *et al. *[48]. Legs of all infected females were used for species identification by Multiplex PCR [49].

### Assessment of the vectorial capacity and host feeding preference of *An. labranchiae*

Vectorial capacity was assessed according to the Macdonald formula [50] revised by Garret-Jones [51]. The experimental variables needed for estimating it were evaluated as follows: the human biting rate (*ma*) by night catches on human bait, the human blood index (HBI) by the origin of the blood meal of the engorged females collected early in the morning in different premises and the parity rate by ovarian dissection [52]. The factors temperature-dependent, such as the length of the sporogonic cycle (*n*) of *P. falciparum *and *P. vivax *and the duration of gonotrophic cycle (*gc*) were calculated according to Macdonald [50]. The host feeding preference *An. labranchiae *was estimated by considering different feeding preference indices in addition to the HBI, i.e. the forage ratio (FR) and the feeding index (FI). FR quantifies vector selection of a particular vertebrate host rather than other available hosts. It was calculated by dividing the percentage of females fed on a given host by the percentage which that host represented in the total census of available animals and humans at the collecting site [53]. FRs significantly > 1.0 indicate a selective bias and values < 1.0 indicate avoidance in favour of other hosts; FRs ≈ 1.0 show neither preference nor avoidance. FI is defined as the observed proportion of females fed on a certain animal host with respect to another one divided by the expected comparative proportion of feeds on these two hosts [54]. This crude index was adjusted by taking in account factors that affect feeding, such as host abundance, their size and their temporal and spatial concurrence with the mosquito species. FI = 1 indicates equal feeding on the two hosts, while smaller or larger values indicate a decrease or increase in feeding on the first host relative to the second, respectively. FI were calculated for each pair of hosts.

The possible relationship between global female abundance and size of the fraction biting man during night catches was also evaluated by the Pearson's statistical test, comparing the data recorded in the same area of site 1 with those from site 3 over a period of 14 years (1995-2008 - our own unpublished data). A coefficient of endophagy of *An. labranchiae *(i.e. the ratio of the number of specimens caught biting indoors versus those caught biting outdoors) was also assessed by the analysis of the retrospective data from human bait catches, performed both outdoors and indoors dwellings in 1994-1996.

### Evaluation of the presence of potential reservoirs of infection

The number of gametocyte carriers that may have been circulating in the territory during the period favourable to malaria transmission (June-October) was obtained by the analysis of the cases of imported malaria in Italy in 2000-2009 (cases confirmed by the Malaria Reference Centre at Istituto Superiore di Sanità), selecting those reported from hospitals located into the study area.

## Results

Results are grouped and showed by parameter adopted for assessing the malariogenic potential of the study area.

### Receptivity

#### Entomological data

Out of a total of 8,006 females belonging to the *An. maculipennis *complex considered in this study, 1,772 (22.1%) were morphologically and molecularly identified at species level. Although at different levels of prevalence and abundance (Table 1), *An. labranchiae *occurred in all the study sites where it represents the dominant species of the *maculipennis *complex, with the exception of site 3 and 5, the rice fields of Val di Merse and the farm of Monte Antico, both located in an hilly area over 300 m a.s.l., where its prevalence accounted for 1-3% and 16% respectively, being predominant *An. maculipennis *s.s. because of the different climate conditions [23,34]. The rice fields of the coastal plain (sites 1-2) remained the most productive areas of *An. labranchiae *(100-500 females/shelter), where it represents 96-98% of the species belonging to the complex. A high prevalence of *An labranchiae *(90-98%), but with lower levels of abundance (range 5-80 females/shelter) was recorded in the remaining study sites, where changes in land use occurred during the last three decades, have contributed to make the territory less favourable to the development of anopheline mosquitoes. A comparison of recent findings with those available for the previous decade showed a reduction of the abundance of *An. maculipennis, s.l*. resting females in the study area of about 75-80%.

#### Seasonality of Anopheles labranchiae

The correlation matrix from the Principal Component Analysis of the weather-based statistical model shows that high temperature, low thermal variability in the last 7 days and slow wind speed are the most relevant variables in determining high abundance categories of *An. labranchiae *adult females for all the three collection sites; the other meteorological variables result in less impact. Skill scores applied to model calibration output for each abundance category, either for the multinomial or the binomial model, shows a very good capacity to discriminate absence from presence in all the three sites: the POD index was always above 90% and FAR below 15%. Slightly lower performances but still satisfactory were obtained for discriminating in site 1 the medium-low abundance from medium-high abundance category: POD resulted equal to 70% and FAR equal to 25%. The BIAS index was in all cases very close to 1, hence it can be inferred that overestimation or underestimation of the model is negligible. Finally, the comparison between predicted and observed values of development season length of *An. labranchiae *adult females (Table 3) resulted in a perfect matching for site 6 both in 2005 and 2006, while for site 4 the predicted length resulted three weeks shorter than the observed one both in 2005 and 2006, for site 1 resulted two weeks longer in 2005 and three weeks shorter in 2006.

**Table 3:** Differences between predicted and observed starting and ending week of development season of *Anopheles labranchiae*

Collection Site	Year	Predicted (week)			Observed (week)			Error (week)		
		Start	End	Length	Start	End	Length	Start	End	Length
**Principina (1)**	**2005**	16	43	27	18	43	25	-2	0	-2
**Ampio (4)**		16	43	27	13	43	30	3	0	3
**Valfragida (6)**		20	43	23	20	43	23	0	0	0

**Principina (1)**	**2006**	20	42	22	18	43	25	2	-1	3
**Ampio (4)**		20	42	22	18	43	25	2	-1	3
**Valfragida (6)**		20	42	22	20	42	22	0	0	0

#### Anopheles labranchiae adult population predictive map

**Figure 2:**
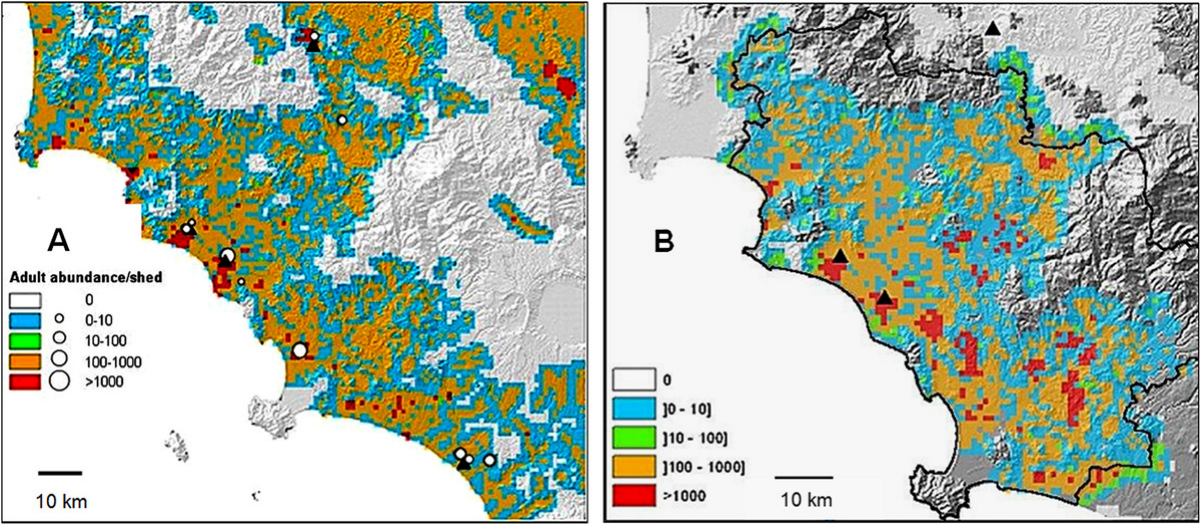
**Predictive weather-based distribution of adult *Anopheles labranchiae *in Maremma, Central Italy**. Adult distribution is related to a basic larval index obtained by elaboration of longitudinal surveillance data from the 4 sites represented by black triangles (sites 1,3,4,6 in Figure 1). Map A shows where and if exist the supposed link between spatial hotspot evidence of anopheline abundance and its proximity to the most relevant breeding sites of the whole study area. White dots represent the real adult abundance recorded in the remaining 6 sites visited sporadically (Table 1) and used to "validate" the map. Map B takes also into account high livestock densities as a weight factor for each pixel of the adult distribution map of the Grosseto Province only (delimited by the black line).

The two predictive distribution maps of adult *An. labranchiae *over the study area are shown in Figure 2. The *An. labranchiae *larval index (based on productivity of *Anopheles maculipennnis sl*. larvae) shows that the occurrence of the species depends on rice paddy areas with a probability of presence > 0.5. The adult index, delineated for each site as the mean value of the larval index within a buffer size (from 500 to 300 m) around the collection site, was then compared to the observed abundance of *An. labranchiae*, in order to find the best buffer size. The best correlation between the adult index and the observed maximum number of *An. labranchiae *captured was detected for a buffer radius of 2000 m. and used to map the predicted abundance of *An. labranchiae *adult populations over the entire study area (Figure 2A). In order to validate these results, the adult indexes generated by the map (colors) in the ten sites reported in Table 1 were compared with the real mean values of females abundance (circles) collected in 2005-2009 in the same sites (Figure 2A). Field collected data matched quite well with those generated by the map, with the exception of site 10 (Diaccia Botrona), where the large surface of the lagoon led to overestimate the adult density, and of site 3 (Val di Merse), where the marked difference between the predictive values of abundance (red color and small circle) is only apparent, because the map considered the total amount of *An. maculipennis s.l*. females, while the small circle refers to the low rate of *An. labranchiae *among the other species of the complex in that area (< 3%). In Figure 2B, the final predictive map of the *An. labranchiae *adult population in Grosseto Province, considering also the livestock presence, showed an increased presence of areas with densities greater than 1,000 adults, particularly in the hilly range.

#### Length of the potential transmission season

**Figure 3:**
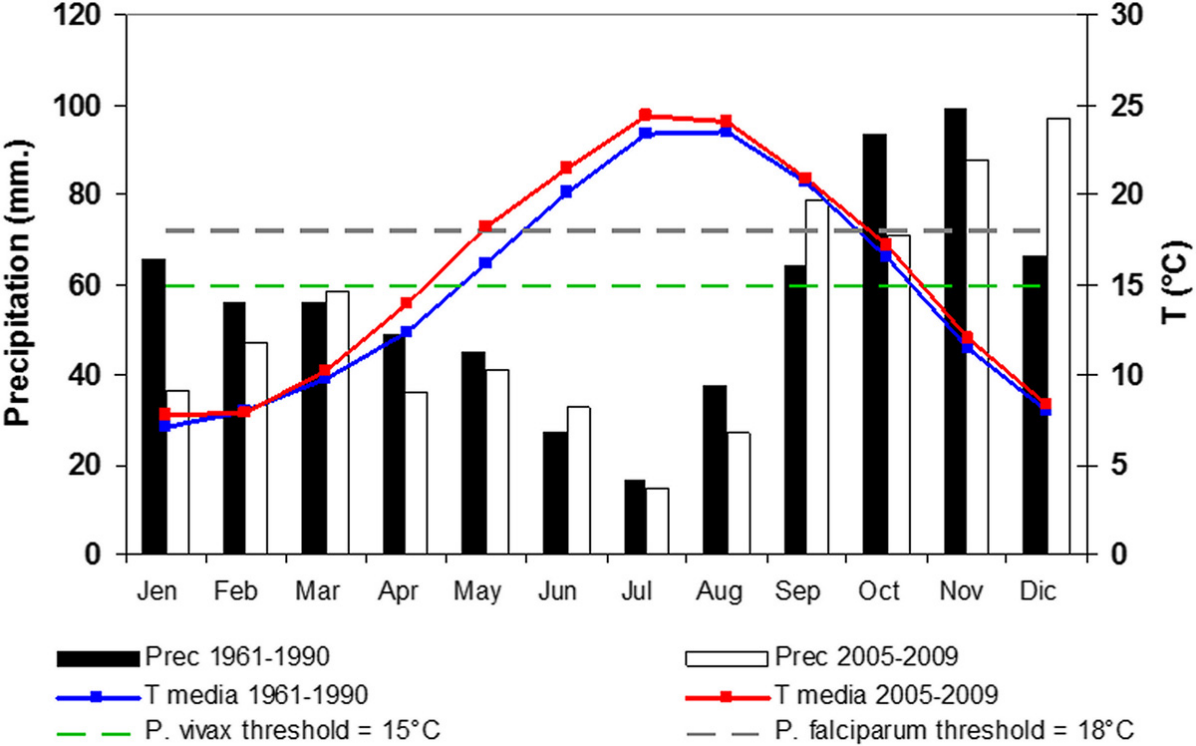
**Comparison of the thermo-pluviometric diagram of the Grosseto province for 1961-1990 and 2005-2009 periods**. White and black histograms represent the mean monthly amount of rainfall (left axe), blue and red line, the monthly mean temperature (right axe) for 1961-1990 and 2005-2009 periods respectively Dotted lines show the minimal temperature required for the development of *Plamodium vivax *and *P. falciparum *that are respectively 15°C and 18°C.

The qualitative comparison of the monthly mean temperature shows that the favourable transmission period for both the *Plasmodium *species extended for one month during 2005-2009 period respected to 1961-1990. In particular during 2005-2009 the start of the favourable transmission period anticipated from May to April for *P. vivax *and June to May for *P. falciparum *(Figure 3).

**Figure 4:**
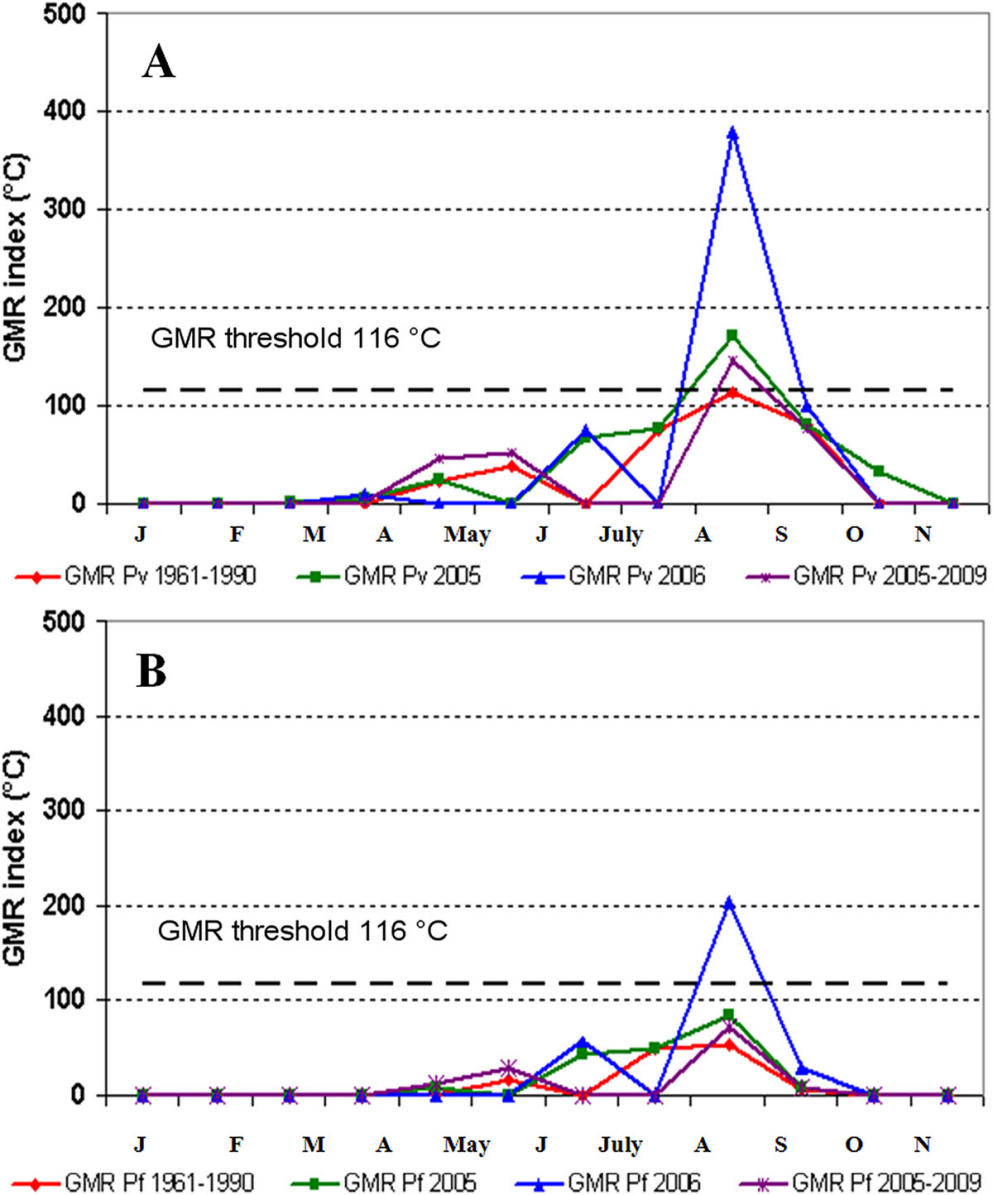
**GMR index monthly evolution diagram for malaria transmission in Maremma region**. Coloured lines represent the period of transmission risk for *Plasmodium vivax *(**A**) and for *P. falciparum *(**B**) in 2005, 2006 and the averaged one in 1961-1990 and 2005-2009 periods.

*Plasmodium *spp. potential transmission risk evaluated through the GMR index calculation [25,37] resulted to be above the threshold, showing (Figures 4A) a potential risk during September for *P. vivax *either in 2005 or 2006 as well as for the 2005-2009 period, while for the climatological period 1961-1990 the index value remaining always below the threshold; for *P. falciparum *the index value exceeds the threshold only in September 2006 (Figures 4B).

**Figure 5:**
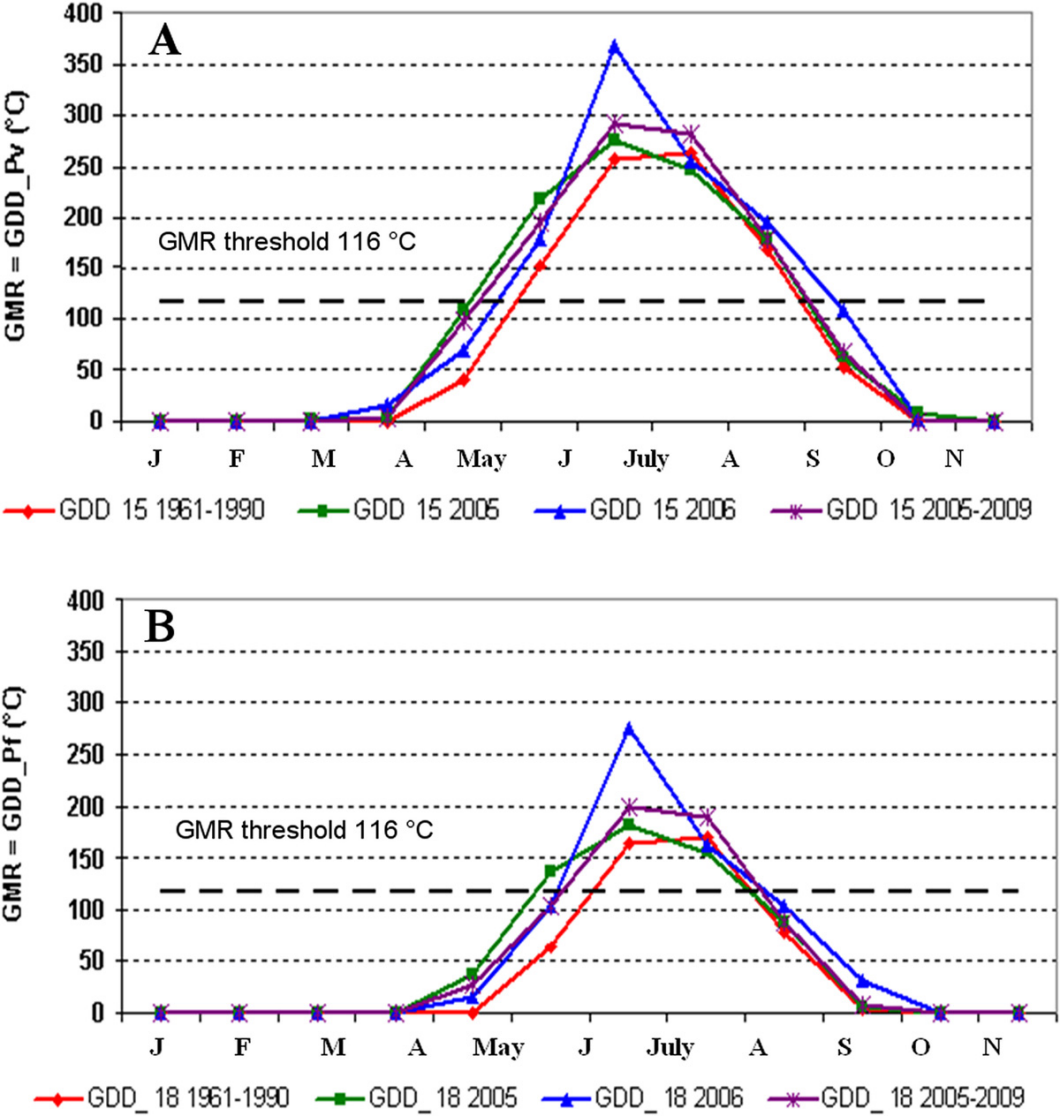
**GMR index monthly evolution diagram sowing the period of malaria transmission in Principina rice fields in 2005, 2006**. Coloured lines represent the period of transmission risk for *Plasmodium vivax *(**A**) and for *P. falciparum *(**B**) in 2005, 2006 and the averaged one in 1961-1990 and 2005-2009 periods. GMR index is equal to GDD when the R/PET ratio is approximately equals to 1.

Concerning the rice field biotope (site 1), it can be stated that R/PET ratio is approximately considered constant and equal to one (see formula in Methods - Evaluation of the length of the possible transmission season) since artificial water supply in rice fields is almost continuous during spring and summer compensating overall evapotranspiration. Hence, GMR index corresponds precisely to GDD calculated with a base temperature of 15°C for *P. vivax *and 18°C for *P. falciparum*. On the basis of this consideration, the favourable transmission risk period for *P. vivax *(Figure 5A) resulted always between June and September, even though either May (for 2005 and for 2005-2009) or October (only for 2006) resulted very close to the threshold. Concerning *P. falciparum*, favourable transmission risk period (Figure 5B) resulted always between July and August, even though June also resulted above or very close to the threshold in 2005-2009.

### Susceptibility

#### Artificial infection

The susceptibility of *An. maculipennis s.l*. population from site 1 to *P. falciparum *was investigated by means of seven attempts of artificial infection with a long established afro-tropical strain of parasite. In 2008, 1,500 resting females were collected on the field and three infection experiments were performed. In total, 25 out of 96 mosquitoes surviving until 8^th ^day, showed an infection prevalence of 26% and a mean number of 0.4 oocyst/mosquito (range 0-4) in the midgut (Table 4). The results of species identification of the infected mosquitoes were: 24 *An. labranchiae *and one *Anopheles melanoon*. In 2009, four infection experiments were performed using 4,500 resting females. Nine out of the 130 surviving mosquitoes dissected in 15^th ^day were found infected (range 0-3 oocysts); in one specimen was observed a possibly mature oocyst. The infection prevalence was 7% and the mean number of oocyst/mosquito was 0.08 (Table 4). It was possible to analyse the head-thorax of 93 specimens, nine with and 84 without oocysts in the midgut, for sporozoites in the salivary glands. Sixty-eight specimens, including the nine with oocysts, were *An. labranchiae*, four *An. melanoon *and 21 not determined. Two specimens of *An. labranchiae*, one with and one without observable oocysts in the midgut, were found to be sporozoite positive.

**Table 4:** Results of the experimental infections of *Anopheles maculipennis *s.l. females from Principina (Grosseto, Italy)

Year	Engorged females	Positive females/total dissected	Prevalence (%)	Mean oocysts/female (int. limit)
**2008**	120	25/96 (after 8 days)	26.0	0.4 (0-4)
**2009**	220	9/130 (after 15 days)	7.0	0.08 (0-3)

In both 2008 and 2009, some infection attempts of F1 first batch of *An. labranchiae *were also carried out but, no infected mosquitoes were detected in these samples. On the whole, fewer than 5% of the field collected females took an infected blood meal, and only 20% of these mosquitoes survived 15 days post infection. In all infection experiments, the *An. gambiae *became infected with oocyst prevalence ranging from 65 to 100%.

#### Vectorial capacity

A study on the assessment of a theoretical vectorial capacity (VC) of *An. labranchiae *in Maremma was performed with the vector population of site 1, the rice-fields of Principina, where the highest abundance of the species was recorded, representing almost all the species of the complex. This was the only site for which it was possible to measure the human blood index (HBI). A summary of the entomological indices necessary for calculating VC is given in Table 5. *An. labranchiae *was the only biting mosquito recorded in 6 nights of collection from June to August, 2005-2006. The landing rate of *An. labranchiae *varied from 6 to 45 landings/man/night. The HBI was determined by the blood analysis of 186 fed females collected in resting sites; 1.6% of the sample (3 specimens contained human blood, 40.3% sheep blood, 24.2% bovine blood, 21% swine blood, 8.1% equine blood and 4.8% fowl blood [23]. The parity rate (P) of *An. labranchiae *from both summer seasons (2005-2006) gradually increased in site 1 as follows: 29.6% (June), 66.7% (July) and 75% (August), 2005 and 10% (June), 46.7% (July) and 69.6% (August), 2006 in site 1 [23]. From this proportion of parity, the probability of daily survival of *An. labranchiae *(*p*) was estimated. In the 2005 summer season, the daily survival rate ranged between 0.66 and 0.92 with an expectancy of infective life for *P. falciparum *ranging between 0.0156 and 0.291, and from 0.0173 and 0.373 for *P. vivax*. In 2006, the daily survival rate varied between 0.518 and 0.74 with expectancy of infective life for *P. falciparum *between 0 and 0.190, and between 0 and 0.259 for *P. vivax*. On this basis, in 2005 the VC for *P. falciparum *ranged from 0.0023 to 0.135 and from 0.0034 to 0.22 for *P. vivax*; in 2006 VC varied from 0 to 0.067 for *P. falciparum *and from 0 to 0.091 for *P. vivax*. The highest VC values were reached in July 2005 for both the *Plasmodium *species.

**Table 5:** Vectorial capacity (VC) of *Anopheles labranchiae *in the rice fields of Principina (Grosseto, Italy), 2005-2006

Parameters considered	Potential transmission season					
	
	Mid June		Mid July		Mid August	
	2005	2006	2005	2006	2005	2006
Average Temperature **(°C)**	25.1	23.6	26.4	26.5	23.7	23.0
Average Relative Humidity **(%)**	50	60	48	60	58	68
Length of gonotrophic cycle (n. days)	3.0	3.5	2.5	2.5	3.5	3.5
*P. falciparum *sporogonic cycle **(n)_*Pf*_**	11	15	12	12	15	16
*P. vivax *sporogonic cycle **(n)_*Pv*_**	10	12	9	9	12	13
Human Blood index **(HBI)**		**0.016**				
Human biting rate **(ma)**	15	12	24	45	6	8
Parity rate % **(P)**	29.6	10.0	66.7	46.7	75.0	69.6
Vector daily survival probability **(p)**	0.666	0.518	0.85	0.74	0.92	0.696
Expectancy of infective life (n. days) **(p^n^)_*Pf*_**	0.0156	0.00005	0.1428	0.0258	0.291	0.190
Expectancy of infective life (n. days) **(p^n^)_*Pv*_**	0.0173	0.00037	0.2323	0.064	0.373	0.259
Expectancy of infective life **(1/-log_e_p)**	2.466	1.52	6.166	3.28	12.16	9.644
Longevity factor **(p^n^/-log_e_p)_*Pf*_**	0.0285	0.00008	0.8805	0.0845	3.5457	1.8356
Longevity factor **(p^n^/-log_e_p)_*Pv*_**	0.043	0.00057	1.432	0.211	4.537	2.505

**VC *P. falciparum***	**0.0023**	**0.0000043**	**0.135**	**0.0240**	**0.097**	**0.067**
**VC *P. vivax***	**0.0034**	**0.000031**	**0.220**	**0.061**	**0.124**	**0.091**

#### Feeding preference and anthropophily of An. labranchiae

Besides HBI, the feeding behaviour of the species, and its rate of anthropophily, was assessed by the calculation of two more feeding indices, FR and FI. Despite sheep being the commonest animal in the study site, the FR for this host resulted the lowest among mammals (< 1.0) indicating a host preference for the other species (Table 6), namely for horses (5.77), pigs (5.0), cattle (3.46) and humans (2.31). A value of 0.55 indicated an avoidance of poultry. A further evaluation was performed using a modified FI that, unlike FR, takes into account the relative size of each host and the contemporary indoor or outdoor occurrence of *An. labranchiae *mosquitoes and hosts during the biting hours. The pair-comparison of human FI with the FI of each mammal host revealed a bias of *An. labranchiae *for humans with respect to the cattle (2.51), horses (1.89) and sheep (1.87) respectively. On the contrary, the FIs for pigs (0.39) and poultry (0.06) revealed that these animals were the preferred source of blood with respect to the humans. The average of the *An. labranchiae *endophagic coefficient, calculated for the three years (1994-1996), was 1.72, 1.28 and 2.02, respectively, showing a preference of the potential vector for biting humans indoors if houses are accessible (i.e. not screened).

**Table 6:** Blood meal sources and forage ratio for *Anopheles labranchiae *in the rice-fields of Principina (Grosseto)

Host	**Total No**.	Mean weight (kg)	Biomass	No. of fed *An. Labranchia*e females	Forage ratio (FR)
Human	2	65	130	3	2.31
Cattle	20	400	8,000	45	3.46
Chicken	25	1.5	37.5	9	0.55
Horse	4	500	2000	15	5.77
Pig	12	90	1,080	39	5.00
Sheep	220	45	9,900	75	0.52

**Figure 6:**
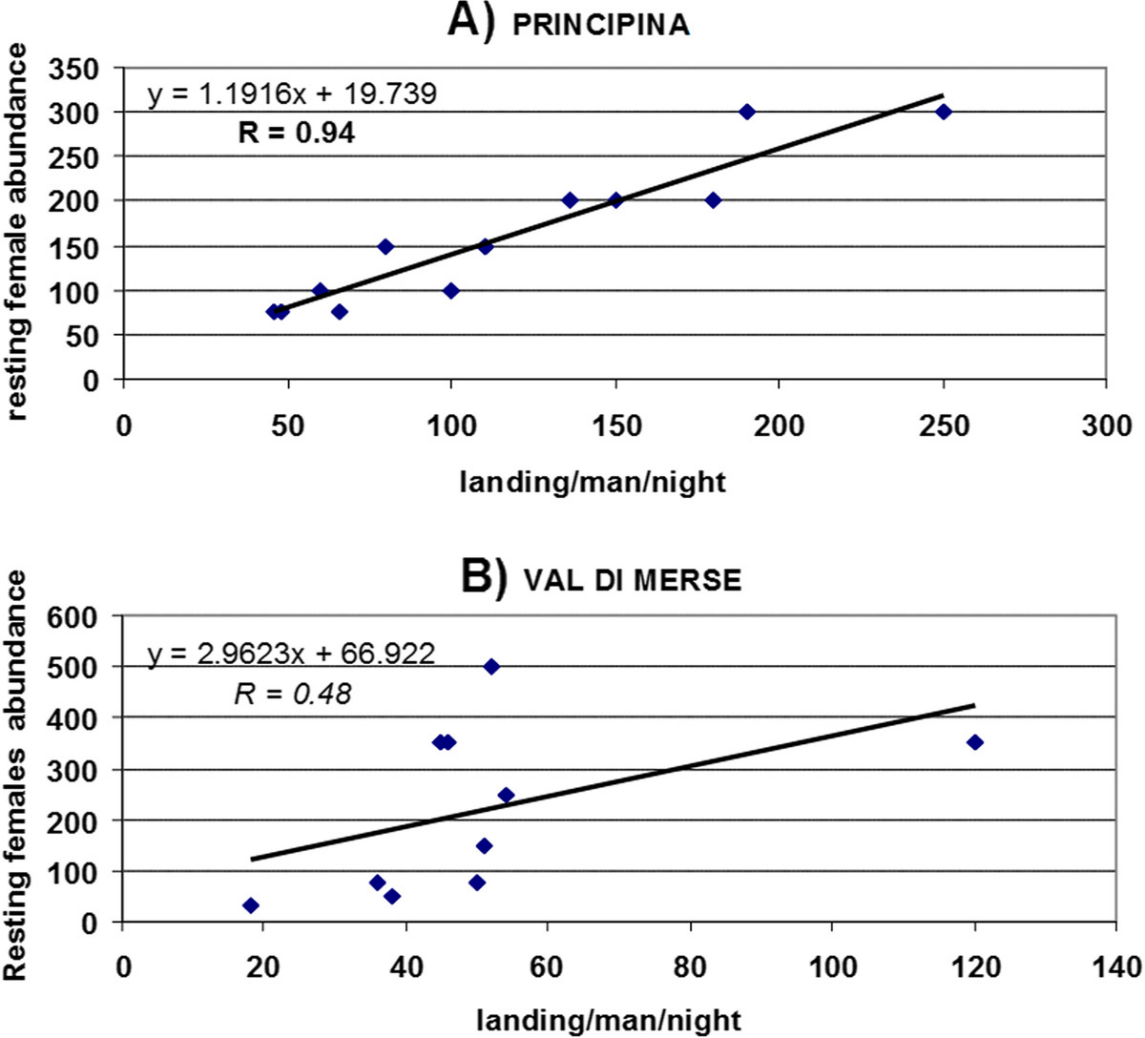
**Scattered plot diagram between adult resting females abundance and number of landing/man/night in two rice-fields sites of Maremma over a period of 14 years (1995-2008)**. Dots represent the number of females collected inside animal shelters in the proximity of the site of Principina (a), where predominant species is *Anopheles labranchia*e and of the Val di Merse site (b), where predominant species is *An. maculipennis *s.s. Pearson coefficient (R) in bold character is significant at 99.9%, italic character means not significant even at 90%.

As further evidence of the more marked anthropophily of *An. labranchiae *with respect to other species of the *An. maculipennis *complex, the correlation between annual data for 1995-2008 of resting females abundance (mainly from pigs and poultry sheds) and number of landings/man/night in sites 1 and 6 was statistically significant at 99.9% (R = 0.94) in site 1 (Figure 6A) where *An. labranchiae *is the predominant species, while in site 3 (Figure 6B), where predominant species is *An. maculipennis *s.s., the correlation was not statistically significant (R = 0.48).

### Vulnerability

#### Gametocyte carrier introduction

A possible introduction of gametocyte carriers from malaria endemic areas able to infect the potential vectors, during the season favourable to malaria transmission, was evaluated (Table 7). In the period 2000-2009, 10 cases resulted from gametocyte carriers coming from Africa, mainly from West Africa (nine cases). Infections were due to *P. falciparum *(five cases), *P. vivax *(two cases) and *Plasmodium ovale *(three cases). Six cases were considered potentially infecting (three due to *P. falciparum*, one to *P. vivax *and two to *P. ovale*) *An. labranchiae *because occurred in the season favourable to malaria transmission (June-October).

**Table 7:** Number of gametocyte carriers circulating in June-October in the study area (2000-2009)

Year	No. of gametocytes carriers/out of national cases	Gametocyte carriers circulating in the study area				
		**No**.	Species	Origin	Month	Potentially infective
2000	132/977 (13%)	1/7	1 *P.f*.	1 Nigeria	1 Sept.	1
2001	126/888 (14%)	2/5	2 *P.f*.	2 Senegal	1 Nov.	0
					1 Feb.	0
2002	106/733 (14%)	4/8	2 *P.f*.	2 Nigeria	2 Oct	2
			2 *P.o*.	1 W. Africa	1 Jul.	1
				1 Nigeria	1 Feb.	0
2003	83/681 (12%)	1/3	1 *P.v*.	1 Senegal	1 Oct.	1
2004	80/673 (12%)	1/2	1 *P.o*.	1 Cameroon	1 Oct.	1
2005	85/637 (13%)	0/3	--	--	--	--
2006	71/630 (11%)	0/6	--	--	--	--
2007	55/575 (10%)	1/5	1 *P.v*.	1 Kenia	1 Jan.	0
2008	81/583 (14%)	0/3	--	--	--	--
2009	75/636 (12%)	0/6	--	--	--	--
Total	894/7,013 (12%)	10/48	--	--	--	6

## Discussion

The presence of potential vectors, the progressive climate increase and the possible introduction of parasite reservoirs raises the concern about the possibility of malaria re-emerging in Italy. The results of previous and present studies and the analysis of historic data showed a continuing receptivity in the Maremma, due to the presence of *An. labranchiae *at all selected sites, despite a marked reduction of the abundance of the vector with respect to the previous 3 decades. Rice fields (site 1, in particular) remained the most productive areas for An. *maculipennis *s.l. However, while in sites1-2 in the coastal plain *An. labranchiae *represents 96-98% of the species belonging to the complex, in the hilly area of site 3 the prevalence of *An. labranchiae *is only 2%. It is noteworthy that in site 3 (where the dominant species is *An. maculipennis s.s*.) *An. labranchiae *historically absent in that area, was first recorded in 2005, possibly indicating a north-eastern expansion of the range of this potential vector.

The vectorial capacity values assessed in site 1 were very low for both *P. falciparum *and *P. vivax*, because a very low size of the HBI (Table 4), in all cases below 0.5, commonly considered as the threshold that characterize a situation of instability or even below 0.02, that should represents the threshold below which the malaria transmission may be interrupted [45]. Nevertheless, it should be considered that the competence of a malaria vector may be strongly affected by environmental factors (temperature and land cover) and by some other traits, related to the trophic activity, such as host feeding preference, which have genetic components [55,56]. There are two critical points in determining vectorial capacity: "ma", that represents human exposure to mosquito bites, may lead to strongly overestimated values [14], and the HBI, that may underestimate vector-human contacts because collections of resting females in the human dwellings are not considered in the current protocols of the entomological surveys in Europe. For these reasons even a small change in accessibility to humans will have a marked impact on the VC values.

Moreover, it should be noted that the daily survival rate p^n ^for the sporogonic development of the parasite in the vector, a factor that strongly affects the Macdonald formula, is a function of temperature.

Hence the rise in temperature appears to represent the most important factor that may influence the receptivity of Maremma. The climatic analysis (Table 2 and Figure 3) outlines a sharp increase of the mean temperature in the study period. From 2005 to 2009 an increase of 0.8°C and 1.2°C respectively for the yearly and the seasonal (May/August) mean temperature or even an increase of 1.2°C and 1.6°C respectively for the yearly and the seasonal maximum temperature was assessed (Table 2). These results are confirmed by recent climatic studies [57-62] which highlight a marked warming and an increase in extreme temperature events in Tuscany, and more generally in Italy, as well as a warming trend elsewhere in the Mediterranean area [63-67]. The potential transmission risk analysis for *P. vivax *and *P. falciparum *evaluated through the GMR index calculation showed in 2005-2009 a larger favourable transmission period during the year than the climatic reference period 1961-1990 (Figures 4, 5 and 6). Climate change scenarios are typically oriented towards higher temperatures but there is greater uncertainty about climate influences on rainfall [68]. However, these uncertainties are irrelevant to the malaria-risk evaluation in the sites of Maremma were the most productive breeding sites are rice-fields that are independent of rainfall. GMR index results obtained with constant R/PET ratio suggest a need for vigilance in the future through surveillance and monitoring activities.

## Conclusions

Although the malariogenic potential of Maremma seemed to be very low, it is worth considering the following points:

i) The study carried out in site 1 showed a high abundance of *An. labranchiae*, and even if the species may appear to be opportunistic in its behaviour, past and present data confirm its ability to bite humans in presence or absence of alternative hosts, indoors as well as outdoors. Moreover, despite the very difficult access to humans, and the very low VC values, it is quite remarkable that to have been found also some *An. labranchiae *gorged on human indicate a little (but not zero) risk of contact human-vector in the area.

ii) Tourism development and changes in land use have resulted often unfavourable to the development of mosquitoes, but in some cases, such as the extension of resorts and holiday farms close to cultivated fields, may be promoting the promiscuity between mosquitoes, the increased availability of non immune humans and of gametocyte carriers.

iii) The very low vulnerability of the study area, due to the scarce presence of gametocyte carriers circulating in the area during the favourable months of the summer may be increased by an unknown number of non regular immigrants entering Italy seasonally as farm labourers (most of them coming from French-speaking West African countries, where malaria is highly endemic).

iv) The general rise in average temperature during the late spring and summer could favour the parasite development, shortening the extrinsic cycle of *Plasmodium spp*, as well as the gonothrophic cycle of the vector and increasing the length of the transmission season.

In conclusion, Maremma, as well as other "at risk" areas recently investigated in the Mediterranean coastal countries, is excluded for the eventual return to a situation of endemic malaria [21,25,29,69-71], while the occurrence of sporadic, isolated cases of introduced *P. vivax *malaria may be considered possible.

## Competing interests

The authors declare that they have no competing interests.

## Authors' contributions

RR conceived, designed and coordinated the study, taking also part in field activities and writing and revising the text. DB, LT and MDL were responsible of field work of the 3 sites of longitudinal surveillance; they also performed molecular identification of mosquitoes, carried out the analysis of entomological data and collaborated to the analysis of the past literature. FS screened morphologically the specimens from the field, being also in charge of the GIS improvement and of the acquisition and management of all field data. MC, IR and GP were involved mainly in rice fields surveillance by fortnightly inspections of the animal shelters, larval collection and in data analysis. AT coordinated the field activities "in loco"(Grosseto) also collecting demographic, economic and historical data. RV GM and AC created the weather-derived mathematical model, evaluated the GMR and performed all statistical analysis; LA and RC implemented the distribution static/predictive maps; IT and AL performed P. falciparum cultures and experimental infections. GLG carried out the molecular analysis for the sporozoites and revised critically the text. AMF was in charge of field activities carried out in upper Latium Region. All Authors participating in writing the manuscript and all of them read and approved the final version.
